# Assessment of early dental arch growth modification with removable maxillary expansion by cone-beam computed tomography and lateral cephalometric radiographs: a retrospective study

**DOI:** 10.1186/s12903-023-03433-w

**Published:** 2023-10-07

**Authors:** Yun Zhang, Jing Yang, Xiaobing Li

**Affiliations:** 1 State Key Laboratory of Oral Diseases & National Clinical Research Center for Oral Diseases, West China School of Stomatology, Sichuan University, Chengdu, 610041 Sichuan China; 2 Department of Pediatric Dentistry, West China Hospital of Stomatology, Sichuan University, Chengdu, 610041 Sichuan China; 3 Department of Pediatric Dentistry, Department of Stomatology, Dandong Central Hospital, Dandong, 118000 Liaoning China

**Keywords:** Dental arch growth modification, Early removable maxillary expansion, Cone-beam computed tomography, Lateral cephalometric radiograph

## Abstract

**Background:**

This study evaluated the skeletal and dental changes of patients brought by early removable maxillary expansion (ERME) treatment to explore the clinical treatment effect of ERME on early dental arch growth modification.

**Methods:**

Subject children aged 6–10 years with a maxillary transverse deficiency received ERME treatment, cone-beam computed tomography (CBCT) and lateral cephalometric radiographs were measured before and after treatment, and statistical differences in the measured items were evaluated with corresponding statistical methods to explore the skeletal and dental changes.

**Results:**

After ERME treatment, there was a statistical increase in the maxillary basal bone arch width, nasal cavity width, maxillary alveolar bone arch width, and maxillary dental arch width. A buccal inclination of the maxillary alveolar bone and a buccal inclination and buccal movement in the alveolar bone of maxillary first molars were found. The maxillary skeletal expansion was statistically greater than the dental expansion. Increases in the mandibular alveolar bone arch width and dental arch width happened after treatment. A decrease in angle ANB and an increase in Ptm-A, U1-SN, U1-PP, L1-MP, and L6-MP were found after treatment. No statistical changes in the growth pattern-related measured items were observed.

**Conclusions:**

ERME could expand the maxillary basal bone arch width, nasal cavity width, maxillary alveolar bone arch width, and maxillary dental arch width. The maxillary skeletal expansion was greater than the dental expansion. Secondary increases in the mandibular alveolar bone and dental arch widths would happen after ERME. ERME would result in a mandibular advancement, a labial inclination of maxillary anterior teeth, and an increase of maxillary sagittal length, and would not change the patient’s growth pattern.

**Trial registration:**

This study was approved by the Institutional Review Board of the West China Hospital of Stomatology, Sichuan University. (WCHSIRB-D-2020–446).

## Background

Maxillary transverse deficiency is a common developmental deficiency in oral examination, of which the etiology is complicated and is related to genetic and environmental factors, including congenital abnormalities, cheek sucking habit, mouth breathing, low tongue position, etc [1–4]. Posterior crossbite is one of the most easily discernible clinical symptoms of maxillary transverse deficiency [5–7], among which unilateral posterior crossbite may result in an increased frequency of reverse chewing cycles and a decreased masticatory efficiency and further causes abnormal facial form and muscle function and asymmetrical mandibular development [8–10]. Maxillary transverse deficiency may lead to anterior crossbite and maxillary dentition crowding [5]. In addition, maxillary transverse deficiency is related to the occurrence of some sagittal malocclusions [11–13] and may affect the dental and maxillofacial growth in the opposite direction because the sagittal and vertical growth continues after the completion of transverse growth [14]. Among the six elements of orofacial harmony proposed by Andrews, "ideal dental arch morphology" is a key element [15]. Therefore, it is of great significance to restore the dental arch morphology and relieve the maxillary transverse deficiency to promote the orofacial harmony of the patients.

Maxillary arch expansion is an effective way to solve the maxillary transverse deficiency [16, 17]. The middle palatal suture of children before or during puberty growth spurt has usually not been completely skeletally fused. In the clinic, orthopedic force and orthodontic force can be applied by maxillary expansion to open the middle palatal suture and expand the width of the maxillary basal bone arch and dental arch, to solve the maxillary transverse deficiency and intercept the occurrence and development of malocclusions or reduce their severity [18–20].

According to the expansion rate, maxillary arch expansion can be divided into rapid maxillary expansion (RME) and slow maxillary expansion (SME), of which the opening rate of the expansion screw is 180°-360° a day and 90° or 180° a week, respectively [21–23]. Compared with RME, SME is softer, closer to the physiological state, and causes less damage to soft and hard tissues. Studies have shown that the overall effects of SME and RME are almost the same, and SME is more stable with more physiological suture response and less recurrence than RME [24–27].

This study intended to make a retrospective study on the dental and skeletal effect of ERME treatment on children with a maxillary transverse deficiency, following the checklist for retrospective studies reported by the ISPOR task force [28]. The skeletal and dental changes were measured in CBCT and lateral cephalometric radiograph before and after ERME treatment in this study, and a comparison of the measured items before and after treatment was carried out to analyze whether there was a statistical change in the bones and teeth after treatment, to explore the clinical treatment effect of ERME treatment on the early dental arch growth modification and provide data support for clinical use of ERME treatment.

## Methods

Forty four children aged 6–10 years with maxillary transverse deficiency who had received ERME treatment in the Department of Pediatric Dentistry, West China Hospital of Stomatology, Sichuan University from 2017 to 2021 were recruited. This study was approved by the Institutional Review Board of the West China Hospital of Stomatology, Sichuan University. (WCHSIRB-D-2020–446).

Inclusion criteria: 1) 6–10 years-old patients during the mixed dentition period; 2) with symptoms of maxillary transverse deficiency including but not limited to the posterior crossbite, the high and arched palatal vault, the uncoordinated maxillary and mandibular arch form, the flared or crowded maxillary anterior teeth, and the deviation of the midline of the mandibular dental arch; 3) with good health, no history of severe craniofacial and maxillofacial abnormalities and trauma, and no history of systemic and genetic diseases; 4) with facial symmetry and coordination, no deformity, and no obvious mandibular retrusion or protrusion; 5) with normal periodontal tissue; 6) had taken CBCT and lateral cephalometric radiographs before and after treatment; 7) with good treatment compliance.

Exclusion criteria: 1) allergic to dental materials such as metal, base resin, etc.; 2) with a history of orthodontic, orthognathic, or prosthodontic treatment; 3) with congenitally missing teeth or premature loss of deciduous teeth; 4) with tooth developmental abnormalities; 5) with untreated upper airway diseases or uncorrected oral habits.

**Fig. 1:**
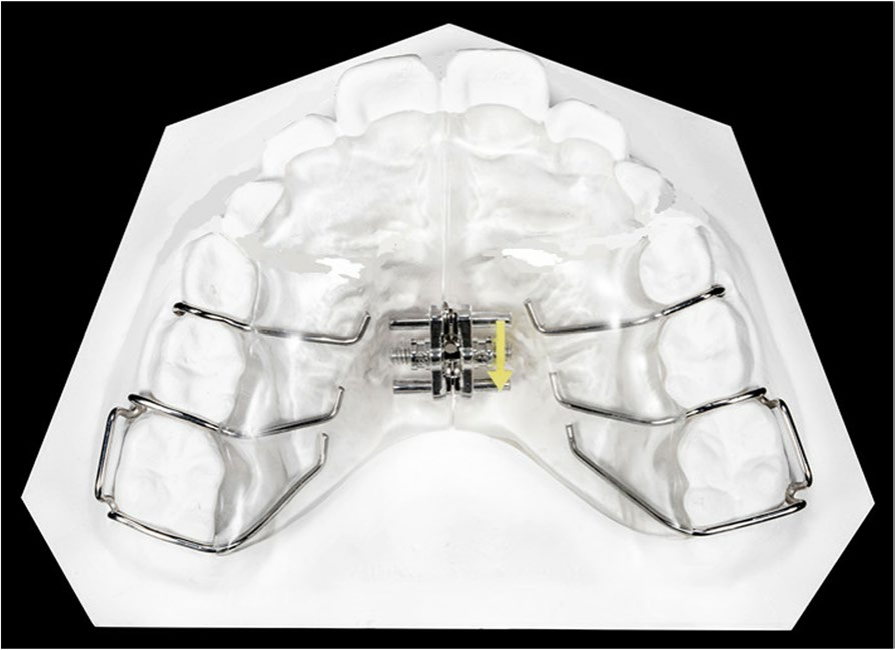
Maxillary removable Schwartz appliance

The patients were treated with a maxillary removable Schwartz appliance. The appliance design **(**Fig. 1) included an acrylic palatal body closely contacting the palatal tissue and gingival tissue and extending to the distal area of the maxillary first molars. Adams clasps were incorporated into the acrylic body at the maxillary first molars, and button clasps were placed into the acrylic interproximal aspect of the first and second deciduous molars for appliance retention. An expansion screw was embedded in the acrylic at the position of the middle palatal suture between the maxillary first and second deciduous molars for arch expansion.

Before treatment (T_0_), the patients’ CBCT (3D Accuitomo 170, Morita, Tokyo, Japan) and lateral cephalometric radiograph (Veraviewepocs, Morita, Tokyo, Japan) were taken with a radiation dose of 129μSv and 1μSv, respectively. During CBCT taking, a lead apron was routinely worn by the patients for radiation protection. The patients were asked to wear the appliance 22 h a day except for meals and tooth brushing during treatment. The expansion screw was activated at 90° a week. The patients revisited the doctor every 1.5 months to check the appliance retention and the treatment effects. The activation was stopped when the palatal cusp of the maxillary first molar was opposite to the buccal cusp of the mandibular first molar, and the appliance was worn for 3 more months for treatment effect retention. CBCT and lateral cephalometric radiograph after treatment (T_1_) were immediately taken after the 3-month retention. No other orthodontic intervention was done during the treatment.

**Fig. 2:**
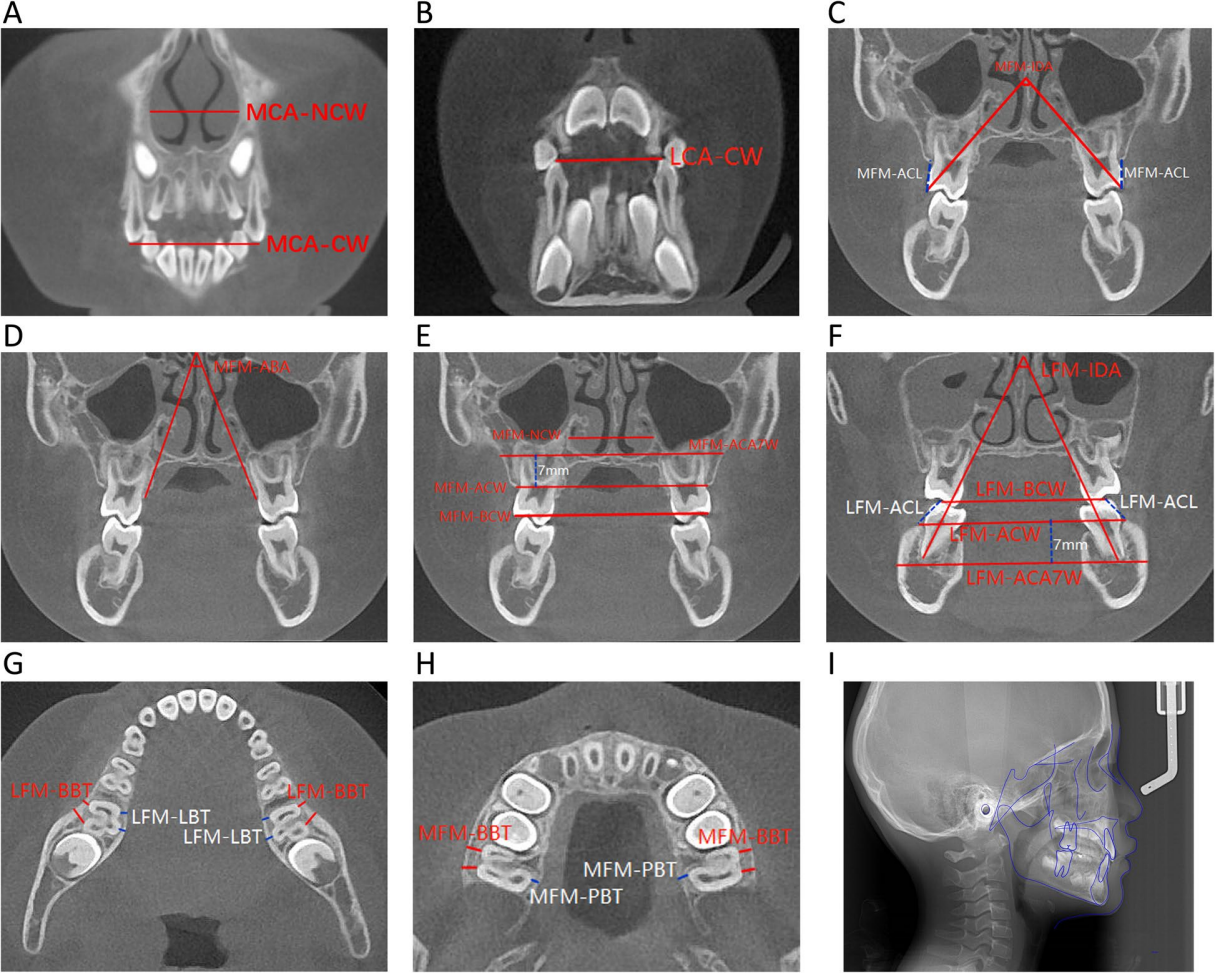
Measured items. **A** MCA-CW (Maxillary canine-cusp width), MCA-NCW (Maxillary canine-nasal cavity width); **B** LCA-CW (Lower canine-cusp width); **C** MFM-IDA (Maxillary first molar-interdental axis angle), MFM-ACL (Maxillary first molar-alveolar crest level); **D** MFM-ABA (Maxillary first molar-alveolar bone axis angle); **E** MFM-BCW (Maxillary first molar-buccal cusp width), MFM-ACW (Maxillary first molar-alveolar crest width), MFM-ACA7W (Maxillary first molar-alveolar crest apically 7mm width), MFM-NCW (Maxillary first molar-nasal cavity width); **F** LFM-IDA (Lower first molar-interdental axis angle), LFM-BCW (Lower first molar-buccal cusp width), LFM-ACW (Lower first molar-alveolar crest width), LFM-ACA7W (Lower first molar-alveolar crest apically 7mm width), LFM-ACL (Lower first molar-alveolar crest level); **G** LFM-BBT (Lower first molar-buccal bone thickness), LFM-LBT (Lower first molar-lingual bone thickness); **H** MFM-BBT (Maxillary first molar-buccal bone thickness), MFM-PBT (Maxillary first molar-palatal bone thickness); **I** Lateral cephalometric radiograph

CBCT images were examined and measured with Mimics software (Mimics 17.0, Materialise, Belgium). The measured items were illustrated in Table 1 and Fig. 2. The lateral cephalometric radiographs (Fig. 2I) were examined and measured with Dolphin Imaging Software (Dolphin Imaging Software 11.8, Dolphin Imaging & Management Solutions, USA).

**Table 1:** Measured items in CBCT

Abbreviation	Measured item	Description	Cross-section
MCA-CW	Maxillary canine-cusp width	The distance between the cusps of bilateral maxillary (deciduous) canines, representing the width of the anterior segment of the maxillary dental arch	The coronal plane of the cusp of the maxillary (deciduous) canine
MCA-NCW	Maxillary canine-nasal cavity width	The distance between the outermost points of the left and right side of the nasal cavity, representing the nasal cavity width at the anterior segment	
LCA-CW	Lower canine-cusp width	The distance between the cusps of bilateral mandibular (deciduous) canines, representing the width of the anterior segment of the mandibular dental arch	The coronal plane of the cusp of the mandibular (deciduous) canine
MFM-IDA	Maxillary first molar-interdental axis angle	The angle formed by the extension line between the buccal cusp and the palatal root apex of the maxillary bilateral first molars, representing the buccal inclination of maxillary first molars	The coronal plane of the widest pulp cavity of the maxillary first molar
MFM-ACL	Maxillary first molar-alveolar crest level	The average distance between the buccal alveolar crest and the buccal cusp of bilateral maxillary first molars	
MFM-ABA	Maxillary first molar-alveolar bone axis angle	The angle between the tangents of the palatal slope of the bilateral maxillary first molar, representing the buccal inclination of the bilateral maxillary alveolar bone	
MFM-BCW	Maxillary first molar-buccal cusp width	The distance between the buccal cusps of bilateral maxillary first molars, representing the width of the posterior segment of the maxillary dental arch	
MFM-ACW	Maxillary first molar-alveolar crest width	The distance between the buccal alveolar crest of the maxillary bilateral first molars, representing the width of the posterior segment of the maxillary alveolar bone arch	
MFM-ACA7W	Maxillary first molar-alveolar crest apically 7 mm width	Make a parallel line of MFM-ACW at the 7 mm apical of MFM-ACW, the distance between the left and right intersection points with maxillary basal bone, representing the width of the posterior segment of the maxillary basal bone arch	
MFM-NCW	Maxillary first molar-nasal cavity width	The distance between the outermost points of the left and right side of the nasal cavity, representing the nasal cavity width at the posterior segment	
LFM-IDA	Lower first molar-interdental axis angle	The angle formed by the long axis of the mandibular bilateral first molars	The coronal plane of the widest pulp cavity of the mandibular first molar
LFM-BCW	Lower first molar-buccal cusp width	The distance between the cusps of bilateral mandibular first molars, representing the width of the posterior segment of the mandibular dental arch	
LFM-ACW	Lower first molar-alveolar crest width	The distance between the buccal alveolar crest of the mandibular bilateral first molars, representing the width of the posterior segment of the mandibular alveolar bone arch	
LFM-ACA7W	Lower first molar-alveolar crest apically 7 mm width	Make a parallel line of LFM-ACW at the 7 mm apical of LFM-ACW, the distance between the left and right intersection points with mandibular basal bone, representing the width of the posterior segment of the mandibular basal bone arch	
LFM-ACL	Lower first molar-alveolar crest level	The average distance between the buccal alveolar crest and the buccal cusp of bilateral mandibular first molars	
LFM-BBT	Lower first molar-buccal bone thickness	The average vertical distance from the buccal edge of the mesial and distal root of mandibular left and right first molar to the buccal bone cortex	The root bifurcation plane of the mandibular first molar
LFM-LBT	Lower first molar-lingual bone thickness	The average vertical distance from the lingual edge of the mesial and distal root of mandibular left and right first molar to the lingual bone cortex	
MFM-BBT	Maxillary first molar-buccal bone thickness	The average vertical distance from the buccal edge of the mesial and distal root of maxillary left and right first molar to the buccal bone cortex	The root bifurcation plane of the maxillary first molar
MFM-PBT	Maxillary first molar-palatal bone thickness	The average vertical distance from the palatal edge of the palatal root of maxillary left and right first molar to the palatal bone cortex

The self-consistency of the examiner was validated by measuring the data of 10 patients twice a week apart in the preliminary experiment and the Kappa value was 0.82, which supported the reliability of the measurements. All statistical analysis was done using SPSS statistical software (IBM SPSS 22.0, Armonk, USA). Shapiro–Wilk test (α = 0.05) was used to test the normality of the data difference (_△_T) of all measured items before and after treatment. Paired t-test for data conforming to normality and Wilcoxon paired rank-sum test for data not conforming to normality were used to test whether there was a statistical difference in the values of each measured item before and after treatment (*P* < 0.05). The null hypothesis of this study was that the measured skeletal and dental values had no statistical differences before and after ERME treatment.

## Results

A total of 44 patients (7.75 ± 1.10 y) were included in this study, including 13 boys (7.46 ± 1.13 y) and 31 girls (7.87 ± 1.09 y). The average treatment time was 9.09 ± 4.94 months.

Table 2 showed that MFM-ACA7W (maxillary first molar-alveolar crest apically 7 mm width) increased by 1.87 mm (*P* < 0.001), and there was no statistical change in LFM-ACA7W (lower first molar-alveolar crest apically 7 mm width) after treatment (*P* > 0.05).

**Table 2:** Measured items before and after the early removable maxillary expansion

Significance	Measured item	T_0_	T_1_	_△_T	*P*-value
**The width of the maxillary and mandibular basal bone arch**	MFM-ACA7W (mm)	62.06 ± 2.35	63.94 ± 2.47	1.87 ± 1.24	0.000***
	LFM-ACA7W (mm)	68.30 ± 3.54	67.68 ± 4.08	-0.62 ± 2.09	0.054
**The width of the nasal cavity**	MCA-NCW (mm)	21.51 ± 2.13	22.22 ± 1.99	0.71 ± 1.98	0.022*
	MFM-NCW (mm)	27.19 ± 1.76	28.26 ± 1.78	1.07 ± 0.76	0.000***
**The maxillary and mandibular alveolar bone arch width**	MFM-ACW (mm)	56.25 ± 2.40	58.47 ± 2.73	2.23 ± 1.46	0.000***
	LFM-ACW (mm)	57.60 ± 2.68	57.98 ± 2.78	0.38 ± 1.16	0.037*
**The buccal inclination of maxillary alveolar bone**	MFM-ABA (°)	39.33 ± 10.36	49.67 ± 10.53	10.34 ± 8.71	0.000***
**The maxillary and mandibular buccal alveolar crest level**	^a^MFM-ACL (mm)	7.52	7.65	0.09	0.199
	^a^LFM-ACL (mm)	7.94	7.99	0.43	0.218
**The maxillary and mandibular dental arch width**	MCA-CW (mm)	32.69 ± 2.23	36.25 ± 2.24	3.56 ± 1.99	0.000***
	LCA-CW (mm)	26.16 ± 2.49	28.64 ± 2.28	2.47 ± 2.52	0.000***
	MFM-BCW (mm)	53.76 ± 2.10	57.31 ± 2.48	3.55 ± 1.61	0.000***
	LFM-BCW (mm)	48.87 ± 2.48	50.85 ± 2.73	1.98 ± 1.70	0.000***
**The buccal inclination of the maxillary and mandibular first molars**	MFM-IDA (°)	55.76 ± 10.42	61.28 ± 12.11	5.52 ± 8.13	0.000***
	LFM-IDA (°)	39.36 ± 9.25	29.74 ± 11.97	-9.62 ± 9.60	0.000***
**The bone thickness of maxillary and mandibular first molar**	LFM-BBT (mm)	3.14 ± 0.91	2.63 ± 0.89	-0.51 ± 0.67	0.000***
	^a^LFM-LBT (mm)	2.15	2.13	-0.06	0.757
	MFM-BBT (mm)	3.45 ± 0.93	2.91 ± 0.75	-0.55 ± 0.81	0.000***
	MFM-PBT (mm)	1.82 ± 0.73	2.01 ± 0.63	0.19 ± 0.51	0.019*
**The buccal and lingual position of maxillary and mandibular first molars in the alveolar bone**	^**a**^LFM-BBT/LFM-LBT	1.50	1.27	-0.26	0.000***
	^**a**^MFM-BBT/MFM-PBT	1.87	1.48	-0.35	0.000***

^a^Wilcoxon paired rank-sum test, median as the value of T_0_, T_1_ and _△_T

^*^P < .05

^***^P < .001

As shown in Table 2, MCA-NCW (maxillary canine-nasal cavity width) and MFM-NCW (maxillary first molar-nasal cavity width) increased by 0.71 mm (*P* < 0.05) and 0.16 mm (*P* < 0.001), respectively. Table 3 indicated no statistical difference in the increasing amount of nasal cavity width on these two coronal planes (*P* > 0.05).

**Table 3:** The difference in the increasing amount of the measured items after the early removable maxillary expansion

Significance	Item	Mean ± SD	*P*-value
**The difference in the increasing amount of MCA-NCW and MFM-NCW**	[(MCA-NCW)^T1^-(MCA-NCW)^T0^] (mm)	0.71 ± 1.98	0.278
	[(MFM-NCW)^T1^-(MFM-NCW)^T0^] (mm)	1.07 ± 0.76	
	[(MFM-NCW)^T1^-(MFM-NCW)^T0^]- [(MCA-NCW)^T1^-(MCA-NCW)^T0^] (mm)	0.90 ± 2.44	
**The difference in the increasing amount of MFM-ACW and LFM-ACW**	[(MFM-BCW)^T1^-(MFM-BCW)^T0^] (mm)	2.23 ± 1.46	0.000***
	[(LFM-BCW)^T1^-(LFM-BCW)^T0^] (mm)	0.38 ± 1.16	
	[(MFM-BCW)^T1^-(MFM-BCW)^T0^]- [(MCA-CW)^T1^-(MCA-CW)^T0^] (mm)	1.85 ± 1.72	
**The difference in the increasing amount of MCA-CW and LCA-CW**	[(MCA-CW)^T1^-(MCA-CW)^T0^] (mm)	3.56 ± 1.99	0.008**
	[(LCA-CW)^T1^-(LCA-CW)^T0^] (mm)	2.47 ± 2.52	
	[(MCA-CW)^T1^-(MCA-CW)^T0^]- [(LCA-CW)^T1^-(LCA-CW)^T0^] (mm)	1.09 ± 2.60	
**The difference in the increasing amount of MCA-CW and MFM-BCW**	[(MCA-CW)^T1^-(MCA-CW)^T0^] (mm)	3.56 ± 1.99	0.979
	[(MFM-BCW)^T1^-(MFM-BCW)^T0^] (mm)	3.55 ± 1.61	
	[(MFM-BCW)^T1^-(MFM-BCW)^T0^]- [(MCA-CW)^T1^-(MCA-CW)^T0^] (mm)	-0.01 ± 2.29	
**The difference in the increasing amount of MFM-IDA and LFM-IDA**	[(MFM-IDA)^T1^-(MFM-IDA)^T1^] (°)	5.52 ± 8.13	0.028*
	[(LFM-IDA)^T0^-(LFM-IDA)^T1^] (°)	9.62 ± 9.60	
	[(MFM-IDA)^T1^-(MFM-IDA)^T0^]- [(LFM-IDA)^T0^-(LFM-IDA)^T1^] (°)	-4.10 ± 11.96	

^*^P < .05

^**^P < .01

^***^P < .001

MFM-ACW (maxillary first molar-alveolar crest width) increased by 2.23 mm (*P* < 0.001) and LFM-ACW (lower first molar-alveolar crest width) increased by 0.38 mm (*P* < 0.05) after treatment as shown in Table 2. Table 3 indicated a statistically greater increase in the alveolar bone arch width in the maxilla than in the mandible (*P* < 0.001).

As shown in Table 2, MFM-ABA (maxillary first molar-alveolar bone axis angle) increased by 10.34° (*P* < 0.001) after treatment.

Table 2 showed no statistical difference in MFM-ACL (maxillary first molar-alveolar crest level) (*P* > 0.05) and LFM-ACL (lower first molar-alveolar crest level) (*P* > 0.05) before and after treatment.

Statistical increases in MCA-CW (maxillary canine-cusp width), LCA-CW (lower canine-cusp width), MFM-BCW (maxillary first molar-buccal cusp width), and LFM-BCW (lower first molar-buccal cusp width) were shown in Table 2. The increased amount was 3.56 mm (*P* < 0.001), 2.47 mm (*P* < 0.001), 3.55 mm (*P* < 0.001), and 1.98 mm (*P* < 0.001), respectively. Table 3 indicated that the increase in the dental arch width on the coronal plane of canines (*P* < 0.001) and first molars (*P* < 0.001) were both statistically greater in the maxilla than in the mandible, and no statistical difference in the increasing amount of dental arch width on these two coronal planes in the maxilla was found (*P* > 0.05).

Table 2 showed that MFM-IDA (maxillary first molar-interdental axis angle) increased by 5.52° (*P* < 0.001) and LFM-IDA (lower first molar-interdental axis angle) decreased by 9.62° (*P* < 0.001) after treatment. Table 3 indicated that the buccal inclination of the first molars after treatment was greater in the mandible than in the maxilla (*P* < 0.05).

A statistical decrease in LFM-BBT (lower first molar-buccal bone thickness) (*P* < 0.001) and MFM-BBT (maxillary first molar-buccal bone thickness) (*P* < 0.001) and a statistical increase in MFM-PBT (maxillary first molar-palatal bone thickness) (*P* < 0.05) was shown in Table 2. For further investigation of the buccal and lingual position of maxillary and mandibular first molars in the alveolar bone, a ratio of LFM-BBT to LFM-LBT and a ratio of MFM-BBT to MFM-PBT were calculated and the difference before and after treatment was tested. Table 2 indicated a statistical decrease in the value of LFM-BBT/LFM-LBT (*P* < 0.001) and MFM-BBT/MFM-PBT (*P* < 0.001).

The expansion of maxillary dental arch width included skeletal expansion and dental expansion. The skeletal expansion was composed of the expansion of the basal bone arch and the buccal inclination of the alveolar bone, which overall manifested as the increase of alveolar crest width. The dental expansion included the buccal inclination and buccal movement of the teeth. As shown in Table 4, the amount of skeletal expansion was 2.23 mm, statistically greater than the 1.32 mm of dental expansion (*P* < 0.05).

**Table 4:** The amount of maxillary skeletal expansion and dental expansion after the early removable maxillary expansion

Item	Mean ± SD	*P*-value
[(MFM-ACW)^T1^-(MFM-ACW)^T0^] (mm)	2.23 ± 1.46	0.018*
[(MFM-BCW)^T1^-(MFM-BCW)^T0^-(MFM-ACW)^T1^ + (MFM-ACW)^T0^] (mm)	1.32 ± 1.46	
[(MFM-ACW)^T1^-(MFM-ACW)^T0^]- [(MFM-BCW)^T1^-(MFM-BCW)^T0^-(MFM-ACW)^T1^ + (MFM-ACW)^T0^] (mm)	0.90 ± 2.44	

^*^P < .05

As shown in Table 5, Ptm-A statistically increased by 0.95 mm (*P* < 0.001), and angle ANB statistically decreased by 0.50° (*P* < 0.01) after treatment. No statistical change was found in SN-MP, FMA, S-Go/N-Me, and ANS-Me/Na-Me after treatment.

**Table 5:** The skeletal measured items in the lateral cephalometric radiograph before and after the early removable maxillary expansion

Measured item	T_0_	T_1_	_△_T	*P*-value
SNA (°)	80.45 ± 2.36	80.40 ± 2.55	-0.05 ± 1.69	0.802
SNB (°)	75.91 ± 2.67	76.35 ± 2.78	0.44 ± 1.87	0.056
ANB (°)	4.54 ± 1.90	4.05 ± 1.72	-0.50 ± 1.24	0.002**
Ptm-A (mm)	41.10 ± 1.95	42.05 ± 2.04	0.95 ± 1.74	0.000***
Ptm-S (mm)	17.09 ± 1.69	17.18 ± 1.60	0.09 ± 1.26	0.560
SN-MP (°)	37.67 ± 4.37	37.90 ± 5.05	0.24 ± 2.95	0.504
FMA (FH-MP) (°)	28.94 ± 4.33	29.50 ± 4.90	0.57 ± 2.64	0.082
S-Go/N-Me (P-A Face Height) (%)	62.69 ± 3.09	62.53 ± 3.17	-0.16 ± 2.07	0.534
ANS-Me/Na-Me (%)	54.62 ± 1.16	54.70 ± 1.25	0.08 ± 1.09	0.528

^**^P < .01

^***^P < .001

Considering that the patients’ growth pattern before treatment may affect the treatment effect on the growth pattern, according to the value of S-Go/N-Me before treatment, the patients were divided into horizontal growth pattern (S-Go/N-Me > 65%), average growth pattern (62% ≤ S-Go/N-Me ≤ 65%), and vertical growth pattern (S-Go/N-Me < 62%). The difference in the growth pattern before and after treatment was separately tested according to the division. Results in Table 6 showed no statistical change after treatment despite the patients’ growth pattern before treatment.

**Table 6:** The growth pattern-related measured items in the lateral cephalometric radiograph before and after the early removable maxillary expansion

Measured item	T_0_	T_1_	_△_T	*P*-value
S-Go/N-Me (horizontal growth pattern) (%)	59.59 ± 1.73	59.84 ± 2.39	0.25 ± 1.89	0.506
SN-MP (horizontal growth pattern) (°)	41.76 ± 3.02	42.10 ± 4.14	0.34 ± 3.01	0.567
FMA (horizontal growth pattern) (°)	32.79 ± 2.93	33.33 ± 4.00	0.53 ± 2.67	0.317
ANS-Me/Na-Me (horizontal growth pattern) (%)	55.16 ± 1.00	54.97 ± 1.37	-0.20 ± 1.08	0.361
S-Go/N-Me (average growth pattern) (%)	63.49 ± 0.80	63.53 ± 2.11	0.03 ± 1.95	0.931
SN-MP (average growth pattern) (°)	36.46 ± 1.77	36.33 ± 3.06	-0.14 ± 2.62	0.786
FMA (average growth pattern) (°)	27.69 ± 2.60	27.78 ± 3.46	0.09 ± 2.33	0.841
^**a**^ANS-Me/Na-Me (average growth pattern) (%)	54.30	54.85	-0.05	0.706
^**a**^S-Go/N-Me (vertical growth pattern) (%)	66.20	65.75	-0.45	0.079
^**a**^SN-MP (vertical growth pattern) (°)	32.80	33.10	0.50	0.615
FMA (vertical growth pattern) (°)	24.29 ± 3.09	25.86 ± 4.22	1.57 ± 3.05	0.076
ANS-Me/Na-Me (vertical growth pattern) (%)	53.93 ± 1.22	54.31 ± 1.56	0.39 ± 1.13	0.223

^a^Wilcoxon paired rank-sum test, median as the value of T_0_, T_1_ and _△_T

Table 7 showed that U1-L1 statistically decreased by 2.38° (*P* < 0.001) and U1-SN statistically increased by 1.81° (*P* < 0.01) after treatment. No statistical change was found in IMPA (*P* > 0.05) and FMIA (*P* > 0.05) after treatment. U1-PP, L1-MP, and L6-MP increased by 0.90 mm (*P* < 0.001), 1.34 mm (*P* < 0.001), and 1.08 mm (*P* < 0.001), respectively.

**Table 7:** The dental measured items in the lateral cephalometric radiograph before and after the early removable maxillary expansion

Measured item	T_0_	T_1_	_△_T	*P*-value
IMPA (L1-MP) (°)	94.05 ± 4.55	94.37 ± 5.29	0.33 ± 4.50	0.554
U1-L1 (Interincisal Angle) (°)	124.57 ± 9.17	122.19 ± 8.60	-2.38 ± 7.25	0.009**
U1-SN (°)	103.71 ± 6.54	105.52 ± 5.90	1.81 ± 5.46	0.008**
FMIA (L1-FH) (°)	57.01 ± 5.51	56.12 ± 5.79	-0.90 ± 3.94	0.065
U1-PP (mm)	24.99 ± 1.72	25.89 ± 1.43	0.90 ± 1.41	0.000***
L1-MP (mm)	34.92 ± 2.36	36.27 ± 2.21	1.34 ± 1.86	0.000***
L6-MP (mm)	26.66 ± 1.61	27.74 ± 1.76	1.08 ± 1.11	0.000***

^**^P < .01

^***^P < .001

Table 8 showed that UL-EP statistically decreased by 0.63 mm (*P* < 0.001) and Z-Angle statistically increased by 1.77° (*P* < 0.01) after treatment.

**Table 8:** The soft tissue measured items in the lateral cephalometric radiograph before and after the early removable maxillary expansion

Measured item	T_0_	T_1_	_△_T	*P*-value
LL-EP (mm)	3.46 ± 1.95	3.27 ± 2.07	-0.19 ± 1.36	0.257
UL-EP (mm)	2.52 ± 1.60	1.89 ± 1.66	-0.63 ± 1.32	0.000***
Z-Angle (°)	60.77 ± 7.15	62.54 ± 6.95	1.77 ± 4.69	0.003**

^**^P < .01

^***^P < .001

## Discussion

In this study, CBCT and lateral cephalometric radiographs of the patients were used to evaluate the treatment effect of ERME. As the evaluation results of CBCT showed, increases in the maxillary basal bone arch width, the nasal cavity width, the maxillary alveolar bone arch width, and the maxillary dental arch width were observed after treatment, and a secondary increase in the mandibular alveolar bone arch width and the mandibular dental arch width happened spontaneously after maxillary expansion. As the evaluation results of the lateral cephalometric radiographs showed, an advancement of the mandible, a labial inclination of maxillary anterior teeth, and an increase in the sagittal length of the maxilla were observed after ERME treatment.

Since there was no standard quantified data on the dental arches of Chinese children, the diagnosis of maxillary transverse deficiency in this study was comprehensively made based on the clinical symptoms of the patients, including uncoordinated maxillary and mandibular dental arch morphology, high and arched palatal vault, crowed maxillary dentition, protruded maxillary anterior teeth, functional deviation of the midline, and so on. Considering the increase of skeletal fusion of the palatal suture with age [29], the removable arch expansion appliance was usually used by the author for children under the age of 10. To treat the maxillary transverse deficiency, a maxillary removable Schwartz appliance was applied in this study. After the activation of the expansion screw, the transverse width of the appliance was increased, resulting in the expansion of the dental arch width and the correction of the maxillary transverse deficiency. The dental arch morphology was restored by arch expansion in the mixed dentition, and the spaces obtained by arch expansion could be used to align the dentition and reduce the tooth extraction ratio of patients with mild to moderate dentition crowding in later orthodontic treatment.

Based on the accuracy of CBCT data, direct measuring and evaluation of CBCT image data before and after maxillary arch expansion treatment is the most commonly used method to evaluate the treatment effect of maxillary arch expansion. This method is simple and fast, and the measurable data are comprehensive, accurate, and three-dimensional. Due to the lack of unified marker points on CBCT, the marker points used in this study referred to several high-quality studies about maxillary arch expansion. The lateral cephalometric radiograph was used as an additional method to evaluate the anteroposterior and vertical changes in the maxilla and mandible.

The results showed that the maxillary basal bone arch width was expanded after the ERME treatment, which was consistent with the results of previous studies on slow maxillary expansion [23, 30]. Referring to the measurement method of Magnusson [11], the widths of the maxillary and mandibular basal bone arches were measured. Although the maxillary expansion appliance acted directly on the bilateral anchorage teeth, the indirect force on the maxillary basal bone could expand the middle palatal suture transversely, thus increasing the maxillary basal bone arch width. While in the mandible, no statistical change in the basal bone arch width was observed after treatment.

ERME treatment evenly expanded the nasal cavity width in the anterior and posterior segments in this study. Referring to the measurement method of Park [31], the nasal cavity widths at the coronal planes of maxillary canines and first molars were measured in this study. The results showed that the nasal cavity widths on these two planes were statistically expanded, which was in accordance with Almeida’s study [30]. Additionally, no statistical difference in the expansion amount at these two planes was found, indicating that the nasal cavity was evenly expanded in the anterior and posterior segments.

Maxillary and mandibular alveolar bone arch widths increased after the ERME treatment, and the increasing amount was greater in the maxilla than in the mandible. To evaluate the effect on the alveolar bone arch of the ERME treatment, the widths of the maxillary and mandibular alveolar bone arches were measured before and after treatment. The increase in the maxillary alveolar bone arch width was in accordance with previous studies [23, 30]. In addition, in the maxilla, the buccal inclination degree of bilateral maxillary alveolar bone was also measured before and after treatment, and a greater buccal inclination degree was found after treatment than before. Therefore, the increase in the maxillary alveolar bone arch width was the overall manifestation of the transverse expansion of the maxillary basal bone and the buccal inclination of the maxillary alveolar bone. Since the mandibular basal bone arch width did not increase after treatment, the increase in the mandibular alveolar bone arch width all resulted from the buccal inclination of the mandibular alveolar bone, which could be a positive treatment effect of the early maxillary expansion.

After the ERME treatment, maxillary and mandibular dental arch widths were expanded at the canine and first molar coronal planes, the increasing amount was greater in the maxilla and the maxillary dental arch width was evenly expanded in the anterior and posterior segments in this study. The maxillary and mandibular dental arch widths at the coronal planes of canines and first molars were measured before and after treatment to evaluate the treatment effect on the dental arches. Results showed that the four widths all increased statistically. The transverse expansion force of the appliance acted directly on the maxillary dental arch, expanding the maxillary dental arch width in the anterior and posterior segments evenly. As the expansion of the maxillary dental arch, the restriction on the transverse growth of the mandibular dental arch was removed, and a secondary increase in the mandibular dental arch width happened, which was an inspiring treatment effect in the clinic. The spontaneous increase in the mandibular intermolar width was consistent with the previous study on the slow maxillary expansion [32]. However, the expansion amount in the mandible was statistically smaller than in the maxilla, because of which a posterior deep overjet could often be observed in the clinic after the ERME treatment. Unilateral posterior crossbite is a severe symptom of maxillary transverse deficiency, which would lead to a unilateral chewing pattern and asymmetrical facial muscles and mandibular bone. According to a previous study by Cutroneo G [9], there was a significantly lower expression in the crossbite side muscle of integrins, which played a key role in regulating the functional activity of muscle and allowing the optimization of contractile forces. The expansion of maxillary dental arch width would improve the posterior crossbite, and further change the chewing pattern and muscle function.

A buccal inclination and a buccal movement of the first molars were found after the ERME treatment in this study, which was similar to the results of Jacob’s research [33]. Buccal inclination and buccal movement of teeth could increase the width of the dental arch, therefore, the buccal inclination degree and the buccal and lingual position in the alveolar bone of the maxillary and mandibular first molars were measured before and after treatment. The results showed that there was a statistical buccal inclination of the first molars both in the maxilla and the mandible, and the buccal inclination was greater in the mandible than in the maxilla, indicating that the increase in mandibular dental arch width might be more attributed to the buccal inclination of the mandibular first molars. In addition, the buccal bone thickness and the ratio of buccal bone thickness to palatal/lingual bone thickness decreased after treatment, indicating that the ERME treatment could result in the buccal movement in the alveolar bone of the maxillary and mandibular first molars.

The skeletal expansion amount was found to be greater than the dental expansion amount in the maxilla in this study. After the ERME treatment, the increase in the dental arch width included the increase of the basal bone arch width, the buccal inclination of alveolar bone, the buccal inclination of teeth, and the buccal movement of teeth. All the above treatment effects were confirmed in this study. The increase in the basal bone arch width and the buccal inclination of the alveolar bone belonged to the skeletal expansion effect and were the most needed arch expansion effect. On the other hand, the buccal inclination of teeth and buccal movement of teeth in alveolar bone belonged to the dental expansion effect and were unstable. As the morphology of the dental arch was limited by the morphology of the basal bone arch, the expansion of the dental arch could not be carried out indefinitely, and the morphology of the two must be coordinated during the arch expansion [34, 35]. In this study, the results showed that the skeletal expansion was 2.23 ± 1.46 mm, statistically greater than the 1.32 ± 1.46 mm of dental expansion, which was inspiring in the clinic.

The alveolar crest height could partly show the periodontal condition of the tooth. Previous studies on maxillary expansion had shown that maxillary expansion treatment may lead to the buccal inclination of teeth, resulting in greater lateral movement of the crown of teeth than that of the root apex, causing the absorption of the alveolar crest and the decrease of alveolar crest height [30, 31, 36]. To explore whether the EMRE treatment would do harm to the periodontal condition of the teeth, the alveolar crest heights were evaluated before and after treatment. As the results showed, no statistical change in alveolar crest height was found after treatment in the mixed dentition in this study. It was speculated that the buccal inclination of teeth caused by the ERME treatment was within a reasonable range that would not cause buccal alveolar crest absorption in the mixed dentitions, or it might result from the strong remodeling ability of alveolar bone in young patients.

A decrease in angle ANB and an increase in Ptm-A were found after treatment in this study. Previous studies had shown that maxillary expansion might cause the extrusion of the palatal cusps of the posterior teeth, resulting in the clockwise rotation of the mandible and the increase of the mandibular plane angle, SNB angle, and the ANB angle [37, 38]. However, the results of this study showed that the ANB angle statistically decreased by an average of 0.5° after treatment. According to the statistical difference in SNA angle and SNB angle before and after treatment, it was inferred that the statistical decrease in ANB angle was mainly caused by the advancement of point B, indicating that the mandible grew spontaneously forward after treatment. The possible reason was that as the expansion of the maxillary dental arch, the restriction on the anteroposterior growth of the mandible was removed, and the mandible continued to grow and develop forward. In addition, the increase in Ptm-A indicated an increase in the anteroposterior length of the maxilla after the ERME treatment in this study, which could be beneficial to Skeletal Class III patients with maxillary sagittal hypoplasia.

In this study, U1-PP, U6-PP, and L1-MP statistically increased after treatment, indicating that the sagittal growth of the maxilla and the vertical growth of the alveolar bone were not limited by the treatment.

The ERME treatment would not affect the growth pattern of patients. SN-MP, FMA, S-Go/N-Me, and ANS-Me/Na-Me were growth pattern-related measured items and were measured before and after treatment. No statistical difference in these items was found before and after treatment, despite the growth pattern of the patients before treatment.

The results showed that angles U1-L1 and U1-SN reduced statistically after treatment, in accordance with the labial inclination of maxillary central incisors in a previous study on slow maxillary expansion [39], while there was no significant change in angle IMPA and FMIA. To sum up, the decrease in the upper and lower central incisor angle after treatment mainly resulted from the labial inclination of the upper anterior teeth. Therefore, in the cases where the labial inclination of the maxillary anterior teeth is not desired, a labial arch must be added to the appliance to limit the labial inclination of the maxillary anterior teeth.

This study was a self-controlled retrospective study comparing the data of CBCT and lateral cephalometric radiographs before and after ERME treatment. The patient samples included in this study were children in the growth and development stage, whose strong development potential could have a great impact on the research data during treatment. However, due to medical ethics considerations, patients with maxillary transverse deficiency could not be divided into blank control groups, so the self-growth of patients other than the effect of ERME treatment could not be measured. Due to the lack of CBCT data for children with normal arch development at the studied age, measurements of the patients in this study before and after treatment could not be compared with those of normal children. In addition, since there were no unified measurement marks for CBCT, the marker points in this experiment were formed by referring to several high-quality relevant research points, the measurement in this study could be further improved after the marker points of CBCT are unified.

## Conclusions

The early removable maxillary expansion could expand the maxillary basal bone arch width, the nasal cavity width, the maxillary alveolar bone arch width, and the maxillary dental arch width. The nasal cavity and maxillary dental arch width could be evenly expanded in the anterior and posterior segments. The maxillary skeletal expansion was greater than the dental expansion. A smaller secondary increase in the mandibular alveolar bone arch width and the mandibular dental arch width would happen after the maxillary expansion. A buccal inclination and a buccal movement of posterior maxillary and mandibular teeth would be caused by ERME treatment, and it would not do damage to the alveolar crest height. In addition, the early removable maxillary expansion would result in an advancement of the mandible, a labial inclination of maxillary anterior teeth, and an increase of maxillary sagittal length, and it would not change the patient’s growth pattern no matter what the patient’s growth pattern was before treatment.

## Data Availability

The datasets used and/or analysed during the current study are available from the corresponding author on reasonable request.

## Abbreviations

ERME: Early removable maxillary expansion
CBCT: Cone-beam computed tomography
RME: Rapid maxillary expansion
SME: Slow maxillary expansion
